# YKL-40-Induced Inhibition of miR-590-3p Promotes Interleukin-18 Expression and Angiogenesis of Endothelial Progenitor Cells

**DOI:** 10.3390/ijms18050920

**Published:** 2017-04-27

**Authors:** Te-Mao Li, Shan-Chi Liu, Ya-Hsin Huang, Chien-Chung Huang, Chin-Jung Hsu, Chun-Hao Tsai, Shih-Wei Wang, Chih-Hsin Tang

**Affiliations:** 1 School of Chinese Medicine, China Medical University, Taichung 40402, Taiwan; leedemaw@mail.cmu.edu.tw (T.-M.L.); jeffrey5991@gmail.com (C.-J.H.); 2 Graduate Institute of Basic Medical Science, China Medical University, Taichung 40402, Taiwan; sdsaw.tw@yahoo.com.tw (S.-C.L.); queena341@hotmail.com (Y.-H.H.); 3 Division of Immunology and Rheumatology, Department of Internal Medicine, China Medical University Hospital, Taichung 40402, Taiwan; u104054003@cmu.edu.tw; 4 Graduate Institute of Clinical Medical Science, China Medical University, Taichung 40402, Taiwan; 5 Department of Orthopedic Surgery, China Medical University Hospital, Taichung 40402, Taiwan; ritsai8615@gmail.com; 6 School of Medicine, China Medical University, Taichung 40402, Taiwan; 7 Department of Medicine, Mackay Medical College, New Taipei City 25160, Taiwan; shihwei@mmc.edu.tw; 8 Department of Biotechnology, College of Health Science, Asia University, Taichung 40402, Taiwan

**Keywords:** YKL-40, IL-18, osteoblasts, angiogenesis, rheumatoid arthritis

## Abstract

YKL-40, also known as human cartilage glycoprotein-39 or chitinase-3-like-1, is a pro-inflammatory protein that is highly expressed in rheumatoid arthritis (RA) patients. Angiogenesis is a critical step in the pathogenesis of RA, promoting the infiltration of inflammatory cells into joints and providing oxygen and nutrients to RA pannus. In this study, we examined the effects of YKL-40 in the production of the pro-inflammatory cytokine interleukin-18 (IL-18), and the stimulation of angiogenesis and accumulation of osteoblasts. We observed that YKL-40 induces IL-18 production in osteoblasts and thereby stimulates angiogenesis of endothelial progenitor cells (EPCs). We found that this process occurs through the suppression of miR-590-3p via the focal adhesion kinase (FAK)/PI3K/Akt signaling pathway. YKL-40 inhibition reduced angiogenesis in in vivo models of angiogenesis: the chick embryo chorioallantoic membrane (CAM) and Matrigel plug models. We report that YKL-40 stimulates IL-18 expression in osteoblasts and facilitates EPC angiogenesis.

## 1. Introduction

Rheumatoid arthritis (RA) is an autoimmune disorder [1,2] characterized by pathological patterns of joint swelling, synovial inflammation, cartilage degradation, pannus formation, and bone erosion [3,4]. While the dynamics of synovial inflammation and cartilage destruction are well-documented, increasing evidence suggests that subchondral bone plays a role in pathogenesis of RA [5,6]. Increasing evidence indicates that osteoblasts in subchondral bone are involved in arthritis development, which regulates many pathological cellular processes including monocyte infiltration, inflammatory cytokine release, and angiogenesis [7,8,9]. An understanding of the normal functions that regulate subchondral bone integrity in arthritis is important for more effective therapeutic strategies in arthritis.

Interleukin-18 (IL-18) is a pro-inflammatory cytokine that is found in autoimmune disorders [4]. High levels of IL-18 are detected in RA synovial fluid and tissue compared with samples from patients with osteoarthritis (OA) and those with other arthropathies [10,11]. During RA pathogenesis, IL-18 promotes the inflammatory process in the RA joint by inducing leukocyte extravasation and acting as a monocyte chemoattractant [12,13]. IL-18 also enhances inflammatory pannus by promoting the migration and angiogenesis of endothelial cells [14,15]. These reports indicate that IL-18 could be an important effector of damage during RA. Moreover, increasing evidence suggests that miRNAs, small, non-coding RNAs that post-transcriptionally regulate gene expression and inhibit RNA stability, play an important role in RA development [16]. Several functions influence how miRNAs affect IL-18 expression [17,18]. For instance, focal adhesion kinase (FAK) has been reported to control the process of angiogenesis [19,20], acting upstream of the PI3K/Akt pathway [21]. However, it remains uncertain as to how FAK, PI3K, and Akt signaling and miRNAs participate in IL-18-regulated angiogenesis.

YKL-40, also known as human cartilage glycoprotein-39 or chitinase-3-like-1, is a pro-inflammatory protein that is highly expressed in RA patients [22]. YKL-40 regulates cell proliferation and survival in human chondrocytes and synoviocytes [23], and facilitates the release of inflammatory cytokines in chronic disease [24,25]. Importantly, YKL-40 is a major secretory product of synovial fibroblasts and articular chondrocytes from patients with RA [26]. Furthermore, adult and fetal chondrocytes, osteoblasts and osteocytes express YKL-40 in OA and RA serum and synovial fluid [27,28], which implies that YKL-40 plays a role in the progression of these diseases.

IL-18 is broadly expressed in osteoblasts from subchondral bone [29,30], which play a critical role in bone remodeling during arthritis [8]. We therefore hypothesized that YKL-40 regulates IL-18-dependent angiogenesis in osteoblasts. We observed in this study that YKL-40 promotes IL-18 upregulation in osteoblasts and subsequently enhances the angiogenic response in endothelial progenitor cells (EPCs). Inhibiting YKL-40 expression diminished the angiogenic response in osteoblast conditioned medium (CM), in both in vitro and in vivo experiments. Our findings suggest that YL-40 facilitates IL-18 expression in osteoblasts and facilitates EPC angiogenesis.

## 2. Results

### 2.1. YKL-40 Promotes IL-18 Production in Osteoblasts and Facilitates EPC Angiogenesis

**Figure 1:**
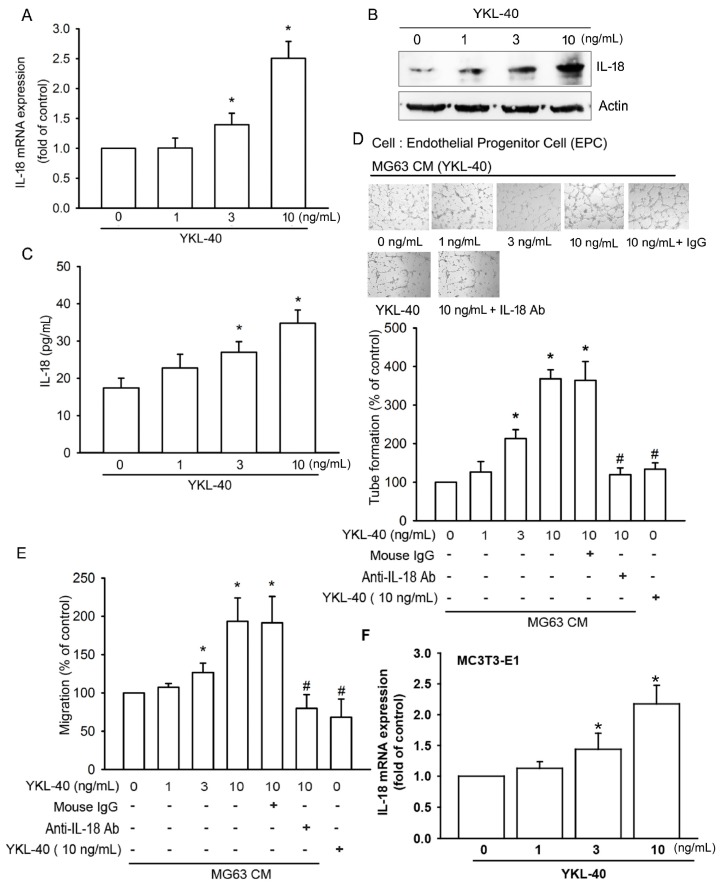
YKL-40 increases IL-18 expression in osteoblasts and promotes endothelial progenitor cell (EPC) angiogenesis. (**A**–**C**) MG-63 cells were treated with prespecified concentrations of YKL-40 (0–10 ng/mL) for 24 h. IL-18 expression was examined by qPCR, Western blotting, and ELISA (*n* = 4 per group). (**D**,**E**) MG-63 cells were treated with YKL-40 (0–10 ng/mL), or pretreated for 30 min with control IgG or IL-18 antibody for 30 min then stimulated with YKL-40 (10 ng/mL) for 24 h. Medium was collected as collected medium (CM). Two hundred microliters of 20% FBS MV2 medium and 150 μL of osteoblast CM was then applied to EPCs. Capillary-like structure formation and in vitro cell migration in EPCs was examined by tube formation and Transwell assay. Direct application of YKL-40 had no effect upon EPC tube formation and migration (*n* = 5 per group). (**F**) MC3T3-E1 cells were treated with prespecified concentrations of YKL-40 (0–10 ng/mL) for 24 h. IL-18 mRNA expression was examined by qPCR (*n* = 3 per group). Results are expressed as the mean ± S.E. * *p* < 0.05 compared with control. ^#^ *p* < 0.05 compared with the YKL-40-treated group.

**Figure 2:**
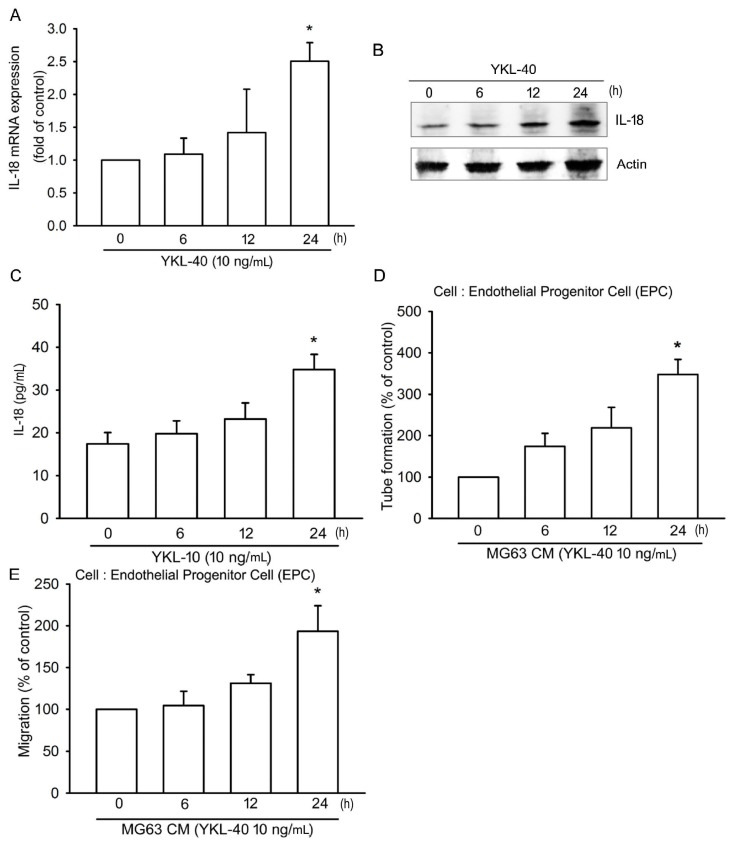
YKL-40 induces IL-18 production and EPC angiogenesis in a time-dependent manner. (**A**–**C**) MG-63 cells were treated with YKL-40 (10 ng/mL) for prespecified time intervals, as indicated. IL-18 expression was examined by qPCR, Western blotting, and ELISA immunoassay procedures (*n* = 4 per group). (**D**,**E**) MG-63 cells were treated with YKL-40 (10 ng/mL) for indicated time intervals. CM was collected and applied to EPCs. Capillary-like structure formation and cell migration of EPCs was examined by tube formation and Transwell assay (*n* = 5 per group). Results are expressed as the mean ± S.E. * *p* < 0.05 compared with control.

We first examined IL-18 production after YKL-40 stimulation. Incubation of osteoblasts (MG-63 cells) with YKL-40 enhanced mRNA expression and protein production of IL-18 in a concentration-dependent manner (Figure 1A–C). Stimulation of EPCs with CM from YKL-40-treated osteoblasts significantly promoted angiogenetic activities (as measured by tube formation and migration assays); these effects were antagonized by IL-18 mAb but not control IgG (Figure 1D,E). However, direct application of YKL-40 had no effect upon EPC tube formation and migration (Figure 1D,E). YKL-40 promoted IL-18 mRNA expression in the osteoblastic cell line MC3T3-E1 (Figure 1F). YKL-40 also promoted IL-18 production, EPC tube formation and migration, in a time-dependent manner (Figure 2), which suggests that YKL-40 induces EPC angiogenesis via IL-18 upregulation.

### 2.2. YKL-40 Promotes IL-18 Expression and Stimulates EPC Angiogenesis through the FAK/PI3K/Akt Signaling Pathway

**Figure 3:**
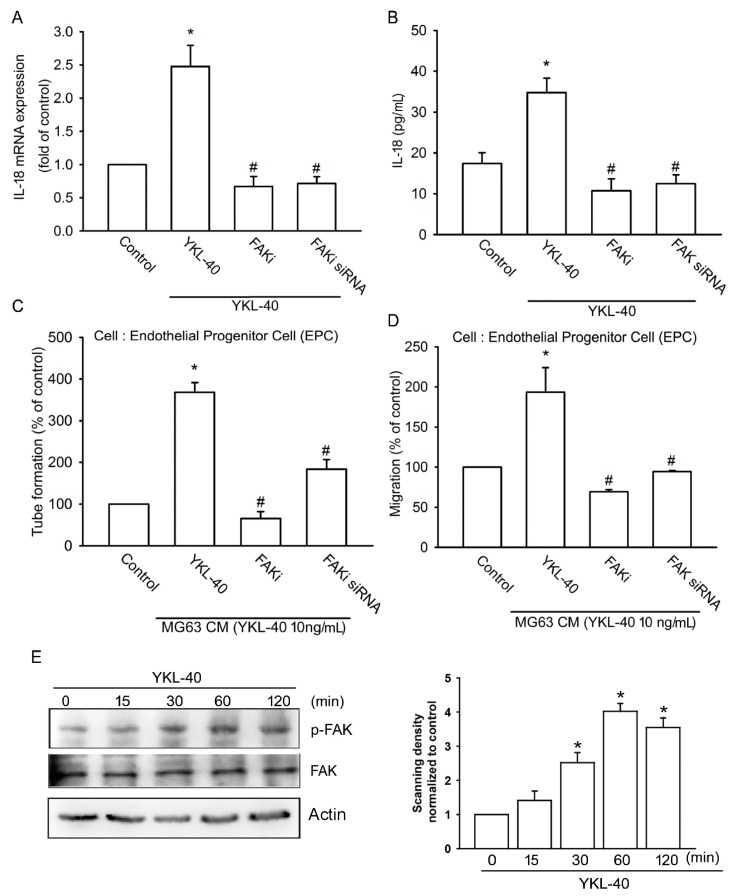
The focal adhesion kinase (FAK) signaling pathway regulates YKL-40-induced increases in IL-18 expression. (**A**,**B**) MG-63 cells were pretreated with a FAK inhibitor (10 μM) or transfected with FAK siRNA for 24 h, then stimulated with YKL-40 for 24 h. IL-18 expression was examined using qPCR and ELISA assays (*n* = 4 per group). (**C**,**D**) CM was collected and applied to EPCs. Capillary-like structure formation and cell migration of EPCs was examined by tube formation and Transwell assay (*n* = 5 per group). (**E**) MG-63 cells were treated with YKL-40 for indicated time intervals, and FAK phosphorylation was examined by Western blotting. FAK phosphorylation in each independent experiment was quantified by densitometry in right panel (*n* = 3 per group). Results are expressed as the mean ± S.E. * *p* < 0.05 compared with control. ^#^ *p* < 0.05 compared with the YKL-40-treated group.

We first examined the effects of FAK upon YKL-40-incuded promotion of IL-18 expression. Pretreatment of osteoblasts with a FAK inhibitor reduced YKL-40-induced IL-18 expression and EPC tube formation as well as EPC migration (Figure 3A–D). Similarly, transfection of osteoblasts with FAK siRNA markedly inhibited all three processes (Figure 3A–D). Incubation of osteoblasts with YKL-40 induced FAK phosphorylation at 15 min, which increased to a maximum level between 60 and 120 min (Figure 3E). These findings demonstrate that FAK activation is involved in YKL-40-stimulated IL-18 expression and EPC angiogenesis.

**Figure 4:**
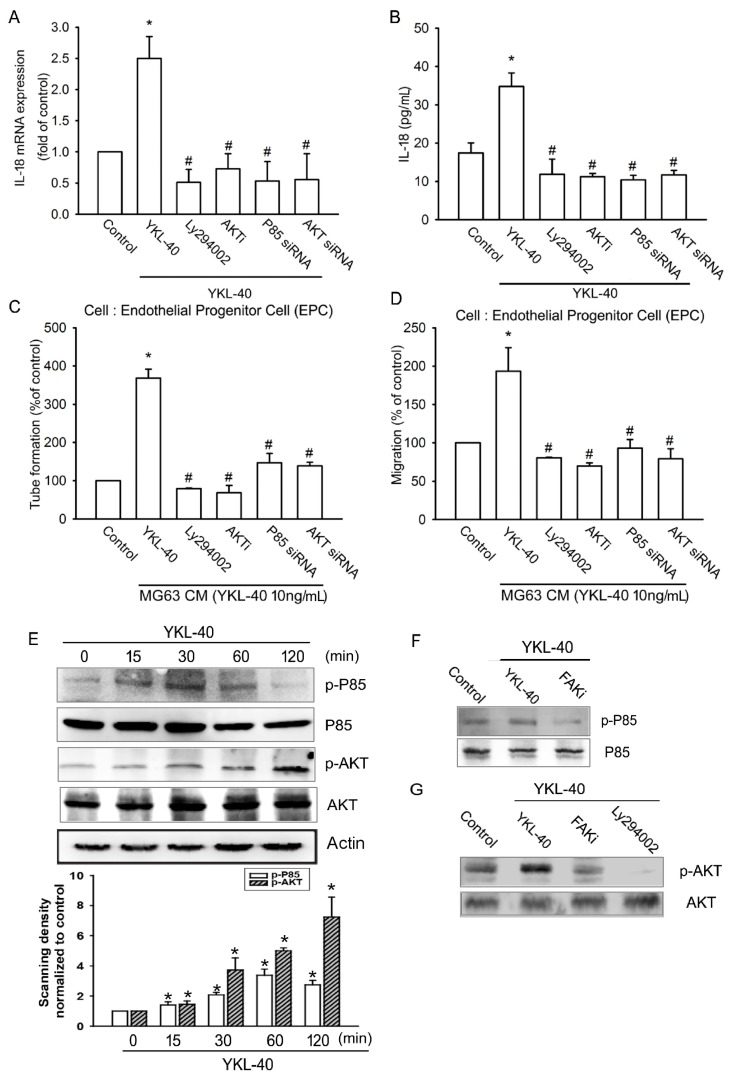
The PI3K/Akt signaling pathway regulates YKL-40-induced increases in IL-18 expression and angiogenesis. (**A**,**B**) MG-63 cells were pretreated with LY294002 (10 μM) and an Akt inhibitor (10 μM) or transfected with p85 and Akt siRNA for 24 h, then stimulated by YKL-40 for 24 h. IL-18 expression was examined by qPCR and ELISA (*n* = 4 per group). (**C**,**D**) CM was collected and applied to EPCs. Capillary-like structure formation and cell migration of EPCs was examined by tube formation and Transwell assay (*n* = 5 per group). (**E**) MG-63 cells were treated with YKL-40 for indicated time intervals, and p85 and Akt phosphorylation were examined by Western blotting. p85 and AKT phosphorylation in each independent experiment was quantified by densitometry (*n* = 3 per group). (**F**,**G**) MG-63 cells were pretreated with a FAK inhibitor or LY294002 for 30 min then stimulated with YKL-40, and p85 and Akt phosphorylation activities were examined by Western blotting (*n* = 3 per group). Results are expressed as the mean ± S.E. * *p* < 0.05 compared with control. ^#^ *p* < 0.05 compared with the YKL-40-treated group.

We next sought to determine whether PI3K/Akt is a downstream event of FAK activation after YKL-40 treatment. We observed that pretreatment of MG-63 cells with the specific PI3K inhibitor LY294002 and siRNA p85 or with an Akt inhibitor and an siRNA inhibited YKL-40-induced facilitation of IL-18 expression and EPC tube formation, as well as migration (Figure 4A–D). We also found that exogenous YKL-40 boosts phosphorylation of PI3K and Akt (Figure 4E). Pretreatment with a FAK inhibitor significantly antagonized YKL-40-increased phosphorylation of p85 (Figure 4F). Pretreatment with either the FAK inhibitor or LY294002 also diminished YKL-40-enhanced Akt phosphorylation (Figure 4G), indicating that YKL-40 acts via the FAK/PI3K/Akt pathway and enhances IL-18 expression in osteoblasts as well as angiogenesis in EPC cells.

### 2.3. YKL-40 Promotes IL-18 Expression via Inhibition of miR-590-3p Expression

**Figure 5:**
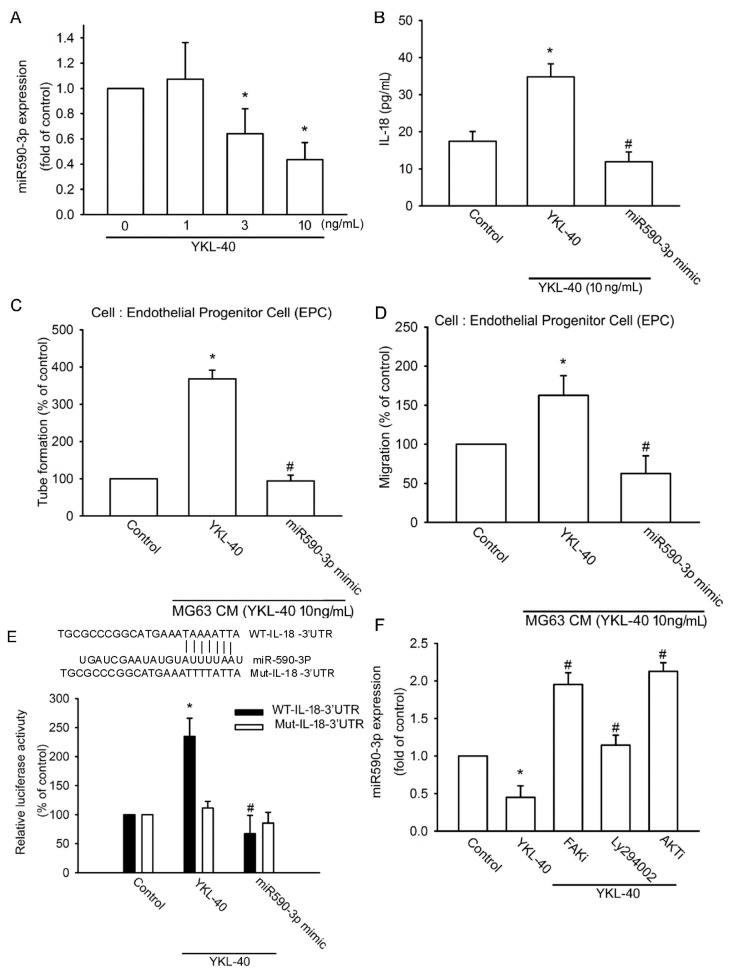
miR-590-3p inhibition regulates YKL-40-induced increases in IL-18 expression. (**A**) MG-63 cells were incubated with YKL-40 for 24 h, and miR-590-3p expression was examined by qPCR (*n* = 5 per group). (**B**) MG-63 cells were transfected with control miRNA or miR-590-3p mimic for 24 h, then stimulated with YKL-40 for 24 h, and IL-18 production was examined by ELISA (*n* = 5 per group). (**C**,**D**) CM was collected and applied to EPCs. Capillary-like structure formation and cell migration of EPCs was examined by tube formation and Transwell assay (*n* = 4 per group). (**E**) MG-63 cells were transfected with the indicated luciferase plasmids for 24 h and stimulated with YKL-40 for 24 h. Luciferase activity was determined (*n* = 4 per group). (**F**) MG-63 cells were pretreated with a FAK inhibitor, LY294002, or an Akt inhibitor for 30 min then stimulated with YKL-40 for 24 h. miR-590-3p expression was examined by qPCR (*n* = 5 per group). Results are expressed as the mean ± S.E. * *p* < 0.05 compared with control. ^#^ *p* < 0.05 compared with the YKL-40-treated group.

Using TargetScan, miRDB, and PicTar software to predict candidate miRNAs that target the 3′UTR region of IL-18 mRNA, we found 20 candidate miRNAs. We observed that miR-590-3p is most strongly downregulated after YKL-40 treatment (data not shown). Exogenous YKL-40 reduced the expression of miR-590-3p in a concentration-dependent manner (Figure 5A). We next applied miR-590-3p mimic to examine whether miR-590-3p regulates YKL-40-mediated IL-18 expression and EPC angiogenesis. Transfection with the miR-590-3p mimic inhibited IL-18 expression facilitated by YKL-40 (Figure 5B) and reduced YKL-40-promoted EPC tube formation and migration (Figure 5C,D), indicating that YKL-40 promotes IL-18 expression and EPC angiogenesis by suppressing miR-590-3p expression.

The wild-type (wt)-IL-18-3′UTR and mutant (mut)-IL-18-3′UTR luciferase plasmids were then examined to determine whether miR-590-3p binds to the 3′UTR region of IL-18 (Figure 5E). Stimulation with YKL-40 enhanced wt-IL-18-3′UTR but not mut-IL-18-3′UTR luciferase activity (Figure 5E). Pretreatment with a FAK inhibitor, LY294002, or an Akt inhibitor prevented YKL-40 inhibition of miR-590-3p expression (Figure 5F). miR-590-3p therefore directly inhibits protein expression of IL-18 via binding to the 3′UTR region of the human IL-18 gene through the FAK/PI3K/Akt pathway.

### 2.4. Inhibition of YKL-40 Reduces Angiogenesis In Vivo

**Figure 6:**
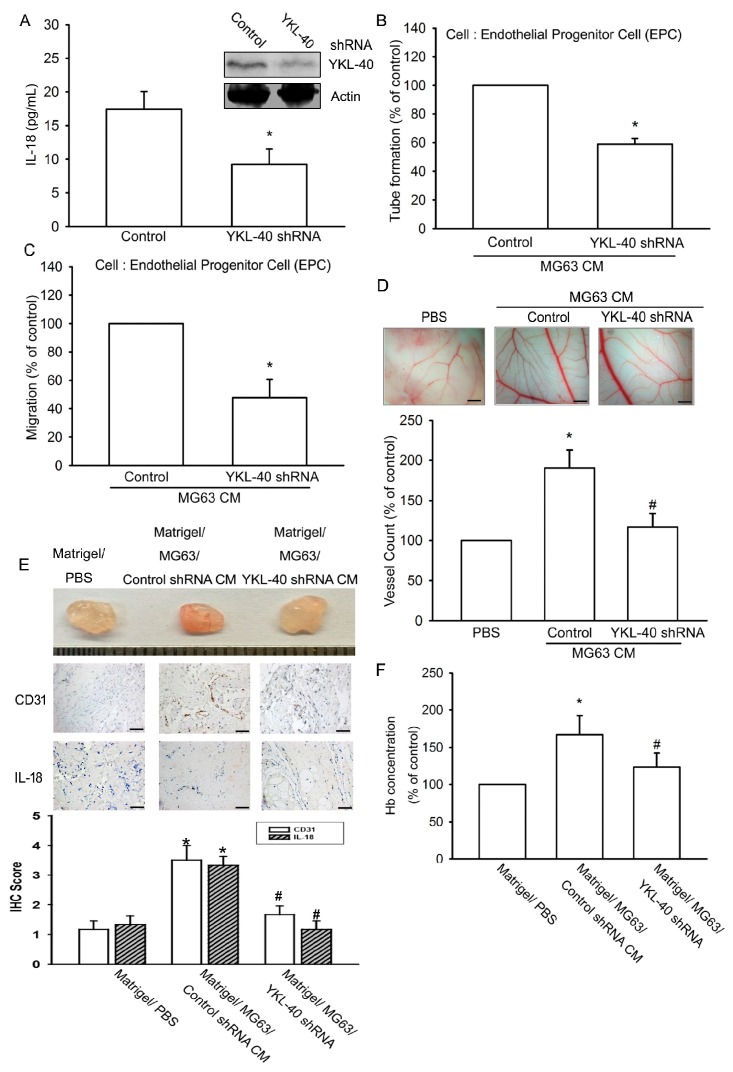
YKL-40 inhibition reduces angiogenesis in vivo. (**A**) MG-63 cells were infected with control or YKL-40 shRNA, then IL-18 and YKL-40 expression were examined by Western blotting and ELISA (*n* = 5 per group). (**B**,**C**) CM was collected and applied to EPCs. Capillary-like structure formation and cell migration of EPCs was examined by tube formation and Transwell assay (*n* = 4 per group). (**D**) CM was also applied for 3-day- to 5-day-old fertilized chick embryos. CAMs were then examined by microscopy and photographed. Angiogenesis was quantified by counting the number of blood vessel branches (*n* = 5 per group). Scale bar = 2 mm. (**E**,**F**) Matrigel plugs containing CM were subcutaneously injected into the flanks of BALB/c male nude mice. After 7 days, the plugs were photographed and hemoglobin levels were quantified by the Drabkin method. Specimens from the plugs were immunostained with antibodies against CD31 and IL-18. Quantification results are shown in the lower panel (*n* = 5 per group). Scale bar = 20 μm. Results are expressed as the mean ± S.E. * *p* < 0.05 compared with control. ^#^ *p* < 0.05 compared with the YKL-40-treated group.

The CAM and Matrigel plug models were used to confirm YKL-40 in vivo enhancement of angiogenesis. Transfection of osteoblasts with YKL-40 shRNA diminished both YKL-40 and IL-18 expression (Figure 6A). In addition, YKL-40 shRNA inhibited tube formation and migration of EPCs induced by osteoblastic CM (Figure 6B,C) and reduced vessel formation in the CAM assay (Figure 6D). Similarly, the Matrigel plug assay revealed YKL-40 shRNA-induced decreases in microvessel formation and hemoglobin, as well as CD31 and IL-18 staining (Figure 6E,F).

## 3. Discussion

RA is an autoimmune disease resulting in swelling of joints, synovial inflammation, cartilage degradation, and bone erosion. Increased angiogenesis is a critical step during RA development, enabling transition of inflammatory cells into joints and providing oxygen and nutrients to RA pannus [15]. IL-18 functions as an angiogenic mediator during arthritis [11], and YKL-40 is a secretory product in arthritic disease [31]; its role in IL-18 production and angiogenesis has been uncertain until now. Here, we identified IL-18 to be a target angiogenic factor of YKL-40. Incubation of osteoblasts with YKL-40 was found to promote IL-18 production and facilitate EPC tube formation and migration. IL-18 mAb treatment diminished CM-mediated EPC angiogenesis. Furthermore, YKL-40 inhibition reduced angiogenesis in vivo. These data suggest that YKL-40 increases IL-18 expression and promotes EPC migration as well as tube formation in osteoblasts through repression of miR-590-3p via the FAK/PI3K/Akt signaling pathway.

Accumulating research suggests that inflammatory cytokines secreted by osteoblasts are important in the joint microenvironment during arthritis [32,33]. These studies highlight that the effect of inflammation on bone is specific to the site of inflammation and dependent on the cytokines present within the local bone microenvironment [32,33]. However, the effects of subchondral bone remodeling in angiogenesis are unclear. This current study found that YKL-40 promotes IL-18 production in osteoblasts and subsequently induces EPC angiogenesis. Knockdown YKL-40 inhibited the expression of angiogenic markers and angiogenesis in vivo. Our evidence indicates that osteoblast-mediated angiogenesis is important during arthritis.

FAK activation is a critical step in the regulation of autoimmune rheumatic diseases [34]. In this study, treatment with a FAK inhibitor or siRNA inhibited YKL-40-induced IL-18 expression. These agents also blocked YKL-40-promoted EPC migration and tube formation. In contrast, YKL-40 treatment of osteoblasts increased FAK phosphorylation, which implies that FAK signaling is essential for YKL-40-induced increases in IL-18 production and EPC angiogenesis. YKL-40 has been reported to activate the PI3K/Akt cascade [35]. Here, both the pharmacologic inhibitors and siRNAs of PI3K and Akt inhibited IL-18 production and EPC angiogenesis. YKL-40 increased phosphorylation of PI3K and Akt, which was diminished by the FAK inhibitor. Therefore, FAK regulation of the PI3K/Akt pathway decreases YKL-40-induced increases in IL-18 expression and EPC angiogenesis.

An earlier report focuses on the effect of miRNAs in gene regulation [36]. It is established that miRNA controls gene expression post-transcriptionally by regulating mRNA [37]. We examined whether miRNAs are mediated in IL-18 production and angiogenesis following YKL-40 stimulation. Our results show that YKL-40 markedly represses miR-590-3p expression in osteoblasts. Transfection of osteoblasts with miR-590-3p mimic diminished IL-18 expression and EPC angiogenesis promoted by YKL-40. In addition, miR-590-3p directly reduced levels of IL-18 protein expression via binding to the 3′UTR of the human IL-18 gene. miR-590-3p is therefore a negative regulator in YKL-40-induced EPC tube formation and migration. Our results show that miRNAs regulate EPC angiogenesis. In conclusion, we found that YKL-40 activates the FAK/PI3K/Akt pathway to increase IL-18 production in osteoblasts and downregulate levels of miR-590-3p, which facilitates EPC angiogenesis.

## 4. Materials and Methods

### 4.1. Materials

Rabbit polyclonal antibodies specific for β-actin, IL-18, p-FAK, FAK, p-p85, p85, p-Akt, and Akt were obtained from Santa Cruz Biotechnology (Santa Cruz, CA, USA). Fetal bovine serum (FBS), DMEM, and other cell culture reagents were obtained from Gibco-BRL Life Technologies (Grand Island, NY, USA). Recombinant human YKL-40 was obtained from PeproTech (Rocky Hill, NJ, USA). miR-590-3p mimic and control miRNA were obtained from GeneDireX (Las Vegas, NV, USA). All pharmacologic inhibitors were obtained from Sigma-Aldrich (St. Louis, MO, USA).

### 4.2. Cell Culture

The human osteoblast-like cell line MG-63 and mouse osteoblastic cell line MC3T3-E1 were obtained from the American Type Culture Collection (Manassas, VA, USA). Cells were maintained in a humidified atmosphere of 5% CO_2_ at 37 °C with DMEM containing 10% FBS and antibiotics (100 U/mL of penicillin G and 100 μg/mL of streptomycin).

Human EPCs were prepared according to our previous protocols [38,39], after obtaining approval from the Institutional Review Board of Mackay Medical College, New Taipei City, Taiwan (reference number: P1000002). Peripheral blood was collected from healthy donors who gave written informed consent. Mononuclear cells were isolated from blood components by centrifugation on Ficoll-Paque plus (Amersham Biosciences, Uppala, Sweden). EPCs were characterized and maintained using methods described in our previous reports [9,40].

### 4.3. ELISA Assay

Osteoblasts were pretreated with pharmacological inhibitors or transfected with siRNA, then stimulated with YKL-40 for 24 h. CM was collected and stored at −80 °C. IL-18 expression in CM was examined using the IL-18 ELISA kit (R&D Systems, Minneapolis, MN, USA), according to the manufacturer’s procedure.

### 4.4. Migration and Tube Formation of EPCs

EPCs (1 × 10^4^) were seeded into the upper chamber of Transwell inserts (Costar, New York, NY, USA). The lower chamber contained 200 μL of 20% FBS MV2 medium and 150 μL of osteoblast CM. After 24 h of migration, cells were fixed in 3.7% formaldehyde solution and stained with 0.05% crystal violet in PBS for 15 min. Migrated cells on the underside of the lower chamber were examined and counted under a microscope.

EPCs (2 × 10^4^) were resuspended in 50 μL of MV2 complete medium and 50 μL of osteoblast CM and then applied to plates pre-coated with 100 μL of Matrigel (BD Biosciences, Bedford, MA, USA). After 6 h, EPC tube formation was photographed and quantified by counting the tube branches.

### 4.5. Quantification of mRNA and miRNA

Total RNA was isolated from MG-63 cells using TRIzol reagent (MDBio, Taipei, Taiwan). mRNA was then reversely transcribed to cDNA using the M-MLV Reverse Transcriptase kit (Invitrogen; Carlsbad, CA, USA). Quantitative real-time polymerase chain reaction (qPCR) was performed with the KAPA SYBR^®^ FAST qPCR Kit (Applied Biosystems, Foster City, CA, USA). For miRNA detection, qPCR was performed using the ABI StepOne™ Real-Time PCR System (Applied Biosystems; Foster City, CA, USA) using the TaqMan MicroRNA Reverse Transcription Kit. Data were normalized to U6 expression [8,41].

### 4.6. Western Blotting

Cellular lysates were isolated according to the methods described in our previous reports [42,43]. The proteins were resolved under SDS-polyacrylamide gel electrophoresis conditions, then transferred electrophoretically onto polyvinyldifluoride membranes (Immobilon, Bedford, MA, USA). Blots were blocked with 4% non-fat milk and incubated with primary antibodies at room temperature for 1 h. After three washes, blots were treated with anti-rabbit or anti-mouse HRP-conjugated secondary antibodies at room temperature for 1 h. Finally, the blots were visualized by enhanced chemiluminescence using Fujifilm LAS-3000 (Fujifilm, Tokyo, Japan).

### 4.7. Plasmid Construction and Luciferase Assay

The 3′-untranslated region of wt IL-18 (wt-IL-18-3′-UTR) contains a miR-590-3p binding site and was constructed into the pmirGLO reporter vector (Promega, Madison, WI, USA) between the Nhel and Xhol cutting sites. The corresponding mutant vector (mut-IL-18-3′-UTR) was obtained using the QuikChange site-directed mutagenesis kit (Stratagene, La Jolla, CA, USA) protocol. Luciferase activity was detected using a luciferase assay system (Promega, Madison, WI, USA). 

### 4.8. Chick Chorioallantoic Membrane Assay

*In vivo* angiogenic activity was evaluated by CAM assay, as described in our previous reports [9,38,39]. Data were examined by microscopy and photographed using a digital camera. Angiogenesis was quantified by counting the number of blood vessel branches. All animal investigations followed approved protocols issued by the China Medical University (Taichung, Taiwan) Institutional Animal Care and Use Committee (103-80-N, 6 November 2014).

### 4.9. In Vivo Matrigel Plug Assay

The Matrigel plug angiogenesis assay was used, as described in our previous reports [9,38,39]. BALB/c male nude mice (National Laboratory Animal Center, Taipei, Taiwan) were subcutaneously injected with Matrigel containing osteoblast CM. On Day 10, Matrigel plugs were harvested and evaluated by the Drabkin’s method (Drabkin’s Reagent Kit, Sigma, St. Louis, MO, USA) to quantify hemoglobin content in blood vessel formation.

### 4.10. Statistical Analysis

All results are presented as the mean ± SEM of at least three experiments. A statistical comparison of two groups was performed using the Student’s *t*-test. Following the two-way ANOVA, post-hoc Bonferroni post-tests were conducted to determine significant differences in statistical comparisons involving more than two groups. In all cases, *p* < 0.05 was considered statistically significant.
